# Microwave Assisted Synthesis of 4-Phenylquinazolin-2(1H)-one Derivatives that Inhibit Vasopressor Tonus in Rat Thoracic Aorta

**DOI:** 10.3390/molecules25061467

**Published:** 2020-03-24

**Authors:** Rafaela Teixeira, Talita Menengat, Gabriel Andrade, Bruno Cotrim, Cristiano Ponte, Wilson C. Santos, Gabriel Resende

**Affiliations:** 1 Programa de Pós-Graduação em Ciências Aplicadas a Produtos para a Saúde, Faculdade de Farmácia, Universidade Federal Fluminense, Niterói 24241-000, RJ, Brazil; 2 Núcleo de Ciência Biomédicas Aplicadas, Instituto Federal do Rio de Janeiro, Rio de Janeiro 20270-021, RJ, Brazil; 3 Núcleo de Ciências Químicas, Instituto Federal do Rio de Janeiro, Rio de Janeiro 20270-021, RJ, Brazil

**Keywords:** microwave irradiation, 4-phenylquinazolin-2(1H)-one, vasorelaxation, endothelium, rat thoracic aorta

## Abstract

Quinazolinones have pharmacological effects on vascular reactivity through different mechanisms. We synthesized 4-phenylquinazolin-2(1H)-one derivatives under microwave irradiation and tested them on the rat thoracic aorta. The prepared compounds **2a–2f** were obtained in about 1 h with suitable yields (31–92%). All derivatives produced vasorelaxant effects with IC_50_ values ranging from 3.41 ± 0.65 µM to 39.72 ± 6.77 µM. Compounds 2c, 2e and 2f demonstrated the highest potency in endothelium-intact aorta rings (IC_50_ 4.31 ± 0.90 µM, 4.94 ± 1.21 µM and 3.41 ± 0.65 µM respectively), and they achieved around 90% relaxation (30 μM). In aorta rings without an endothelium, the effect of compound 2f was abolished. Using the MTT assay to test for cell viability, only compound 2b induced cytotoxicity at the maximum concentration employed (30 µM). The results show that vasorelaxation by 4-phenylquinazolin-2(1H)-one derivatives might depend on the activation of a signalling pathway triggered by endothelium-derived factors.

## 1. Introduction

**Figure 1:**
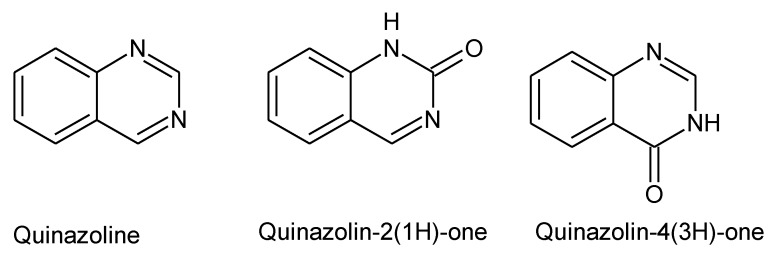
Quinazoline and quinazolinone structures.

Heterocyclic drugs make up over 60% of major retail medicines for the pharmaceutical market [1,2]. Considering the heterocyclic quinazolines and quinazolinone-containing compounds, they have already shown pharmacological relevance with broad therapeutic potential [3]. Hence, several quinazoline and quinazolinones have been applied in the chemical and pharmaceutical fields, and many of them have already been approved as medicines [4]. Quinazolinones are heterocyclic chemical compounds with two structural isomers, namely quinazolin-2(1H)-one and quinazolin-4(3H)-one (Figure 1); the latter is the most investigated in the literature [5]. 

Quinazoline derivatives have also demonstrated antihypertensive effects, such as the class of the alpha 1-adrenergic (α1) receptor antagonists used in the treatment of hypertension [6,7]. Drugs such as prazosin or terazosin act on vascular smooth muscle receptors, leading to vasodilation and a consequent reduction in peripheral vascular resistance; these medications are used for the treatment of benign prostatic hyperplasia (BPH) [8]. Concerning their antihypertensive effects, quinazolines have shown activity on vascular reactivity through different mechanisms such as the modulation of T-type calcium channels [9], angiotensin converting enzyme (ACE) [10] and phosphodiesterase-5 (PDE-5) [11]. Indeed, several therapeutic activities of quinazolines and their derivatives have been reported in the literature, including antimicrobial [12], antitumor [13,14] and anticonvulsant [15]. In addition, interest in the synthesis and biological activity of quinazolinones and quinazolines has contributed to the growing number of new patents for such molecules in recent years [4].

Thereby, considering all this knowledge, the existence of several reports on vascular activity attributed to quinazolines and, notably, few studies that focus on the biological effects of the isomer quinazolin-2(1H)-one, we decided to investigate the putative effects of its derivatives on the rat thoracic aorta. In the present investigation, we synthesized 4-phenylquinazolin-2(1H)-one derivatives synthetized under microwave irradiation and tested them for their vasorelaxation properties using experimental protocols on the rat thoracic aorta.

## 2. Results and Discussion

### 2.1. Chemistry

**Scheme 1:**
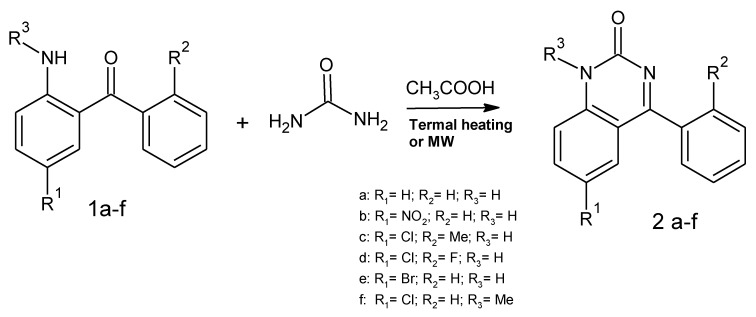
Synthesis of 4-phenylquinazolin-2(1H)-one derivatives.

Several methodologies for the 4-phenylquinazolin-2(1H)-one scaffold have been described in the literature; however, they require prolonged reaction times [16,17] or expensive reagents [18,19,20,21]. Most of the time heating using microwave radiation allows one to drastically reduce times and increase the yield of reactions. Microwave radiation provides more efficient heating since it is generated internally by direct absorption of microwave radiation by polar molecules present in the reaction mixture (for example solvents, reagents and catalysts) [22,23]. In order to improve the existing synthesis protocol, a series of 4-phenylquinazolin-2(1H)-one derivatives were synthetized using a conventional methodology and microwave (MW) irradiation with urea and substituted 2-aminobenzophenones as the preliminary compounds (Scheme 1). The results are recorded in Table 1.

Table 1 shows that the methodology using microwave heating provided higher yields and required less reaction time compared with the traditional methodology. The compounds **2a**–**f**, except compound **2b**, were obtained in less than 1 h and obtained with good yields (63–92%). Compound **2b**, which has an electron withdrawing group (nitro group), had very poor yield with the traditional methodology (16%), but this was increased using the microwave methodology (31%).

### 2.2. Biological Evaluation

**Figure 2:**
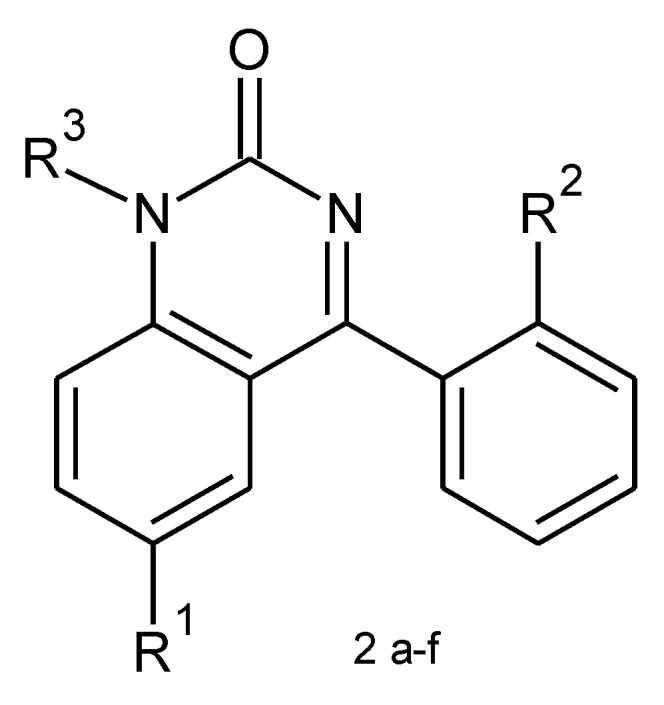
Structure of 4-phenylquinazolin-2(1H)-one derivatives.

**Figure 3:**
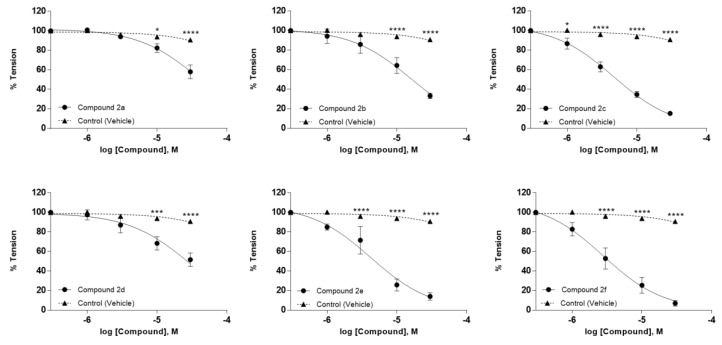
Concentration-response curves for compounds **2a**–**f** and control (vehicle: DMSO) in intact endothelium aortic rings pre-contracted with Phe 1 µM. Cumulative concentrations (1 µM, 3 µM, 10 µM and 30 µM) were added to the Phe contraction plateau. DMSO was employed at the same volume used to dissolve the compound stock solution (maximum final concentration at 0.3%). All data are expressed as mean ± standard error of the mean (SEM) (n = 4–5). (* *P* < 0.05; *** *P* < 0.001; **** *P* < 0.0001 compound vs. control).

All tested compounds induced concentration-dependent relaxation (IC_50_ ranging from 3.41 ± 0.65 µM to 39.72 ± 6.77 µM) in endothelium-intact aorta arterial rings pre-contracted with Phe 1 µM (Table 2, Figure 2 and Figure 3). The cumulative addition of increasing concentrations of the studied compounds potentiated the observed vasorelaxant effect. Compound **2f** was found to be the most potent in endothelium-intact vessels (IC_50_ = 3.41 ± 0.65 µM), while compounds **2a**, **2b** and **2d** showed the lowest potency in the endothelium-intact aorta at the concentrations employed in the present study. Additionally, compounds **2c**, **2e** and **2f** demonstrated the highest potency with an IC_50_ of 4.31 ± 0.90 µM, 4.94 ± 1.21 µM and 3.41 ± 0.65 µM, respectively, and they achieved around 90% relaxation at the highest concentration of 30 μM in endothelium-intact aorta rings. Thus, we decided to test compound **2f** at lower concentrations (30–300 nM) in endothelium-intact and endothelium-denuded aorta rings.

**Figure 4:**
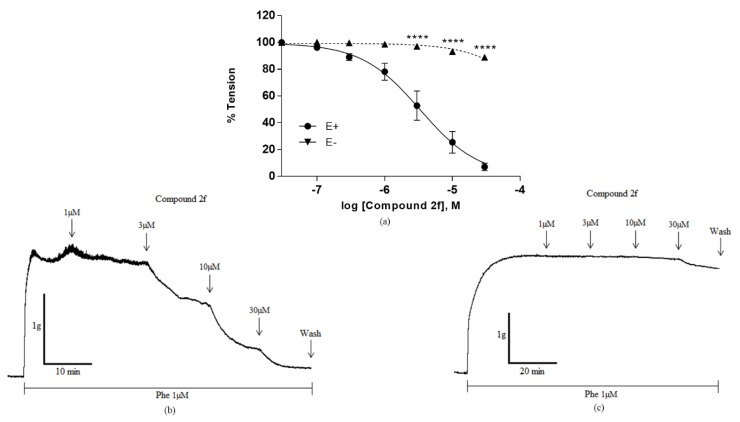
Concentration-response curves of compound **2f** in endothelium-intact (E+) and endothelium-denuded (E−) aortic rings pre-contracted with Phe 1 µM. (**a**) Cumulative concentrations (30 nM, 100 nM, 300 nM, 1 µM, 3 µM, 10 µM and 30 µM) were added to the Phe contraction plateau. (**b**) Representative record of vasorelaxation induced by the cumulative addition (1 µM, 3 µM, 10 µM and 30 µM) of compound **2f** in endothelium-intact (E+) aortic rings pre-contracted with Phe 1 µM. (**c**) Representative record of vasorelaxation induced by the cumulative addition (1 µM, 3 µM, 10 µM and 30 µM) of compound **2f** in endothelium-denuded (E−) aortic rings pre-contracted with Phe 1µM. All data are expressed as mean ± standard error of the mean (SEM) (n = 4). (**** *P* < 0.0001 E+ vs. E−).

Preliminarily, we decided to investigate the effect of compound **2f**, as the most effective in the series, in an experimental model without the endothelium to assess its possible involvement on the observed vasorelaxation. A representative record of cumulative concentrations of compound **2f** (1 μM, 3 μM, 10 μM and 30 μM) on aortic rings endothelium-intact and endothelium-denuded pre-contracted with Phe 1 μM are shown in Figure 4. Phe induced a steady tone in both preparations that were greater in endothelium denuded rings. The results demonstrate that vasorelaxation was abolished in the absence of the endothelium.

**Figure 5:**
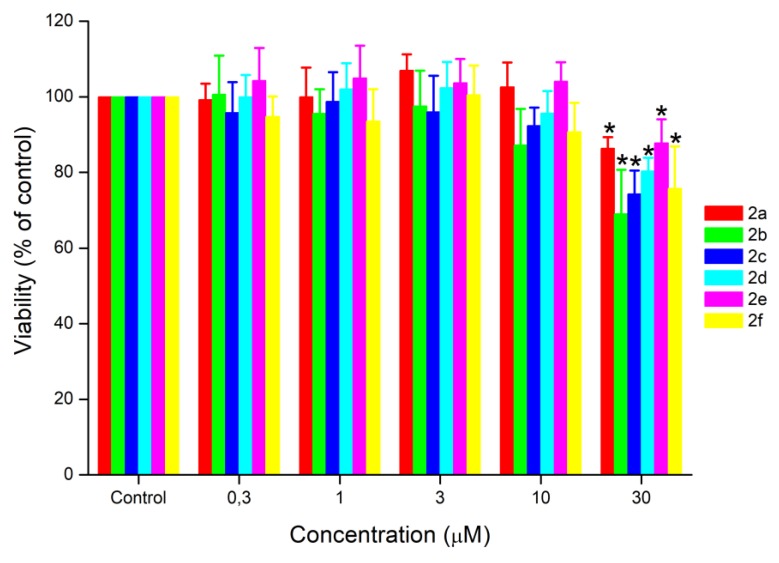
Cell viability assessed by the MTT method on the VERO cell line after 48 h of exposure to the compounds. The cells were exposed to different concentrations of the compounds (0.3 µM, 1 µM, 3 µM, 10 µM and 30 µM). All data are expressed as mean ± standard error of the mean (SEM) (n = 22–25). (DMSO maximum final concentration at 0.3%) (* *P* < 0.05, compound vs. control).

Based on the results of the MTT assay (Figure 5), it was observed that none of the compounds induced a significant difference in the percentage of cell viability at the lowest concentrations used (0.3–10 µM). Although all six compounds (**2a–2f**) showed a significant difference at the highest concentration of 30 µM, it is important to note that the percentage of living cells in this case was above 70%, except for compound **2b** (69.05%). According to ISO 10993: 5 (2009), samples that reduce cell viability values below 70% should be considered cytotoxic. Therefore, only compound **2b** could be considered cytotoxic at the concentration of 30 µM. In addition, we highlight that these compounds in general had IC_50_ values below 30 µM.

The main findings show that compounds **2a**–**2f** were able to relax the aorta. Furthermore, the results indicate an effect directly related to the presence of the endothelium for the **2f** analogue. Therefore, it is possible that vasorelaxation is dependent on the modulation of some signalling pathway triggered by endothelium-derived factors (EDFs) [24]. It is noteworthy that nitric oxide (NO) is one of the principle vasodilators related to tone control in most vessels, so our results with the **2f** derivative suggest the possibility of the involvement of NO in the observed vasorelaxation [25]. However, other physiological mechanisms that play a role in the activity of the vascular smooth muscle contractile machinery cannot be disregarded [26,27]. Consequently, some other protocols must be tested to study the influence of other pathways on the observed response.

As is broadly known, endothelial dysfunction is one of the relevant features in the pathophysiology of hypertensive disorders such as hypertension [28]. Therefore, substances that directly affect endothelial functional regulation such as the quinazoline derivatives studied in the present paper may have significant potential in the treatment of circulation diseases [29].

## 3. Materials and Methods

### 3.1. General Information

The reactions were carried out in a 300-W CEM Discover focused microwave reactor (CEM Microwave Technology Ltd., Buckingham, UK). Reagents were purchased from Sigma-Aldrich Brazil (São Paulo, SP, Brazil) or donated by Nortec Química S/A (2-aminobenzophenones **1b**–**f**) and were used without further purification. Column chromatography was performed with silica gel 60 (Merck 70–230 mesh, RJ, Brazil). Analytical thin-layer chromatography (TLC) was performed with silica gel plates (Merck, TLC silica gel 60 F254), and the plots were visualized using UV light or aqueous solutions of ammonium sulphate. Yields refer to chromatographically and spectroscopically homogeneous materials.

### 3.2. Chemistry

#### 3.2.1. General Procedure for the Preparation of **2a**–**f**

##### Conventional

A mixture of 2-aminobenzophenone **1a**–**f** (1 mmol) and urea (15 mmol) in glacial acetic acid (10 mL) was stirred at 140 °C and the progress of the reaction was monitored by TLC. After completion of the reaction, the reaction mixture was filtered, and the precipitate was washed with water. The physical and spectroscopic data were previously reported in the literature for **2a**, **2e** and **2f** [30]; **2b** and **2d** [31]; **2c** [18].

##### Microwave Irradiation

A mixture of 2-aminobenzophenone **1a**–**f** (1mmol) and urea (15 mmol) in glacial acetic acid (10 mL) was irradiated in a sealed tube at 140 °C (200 w) for 30–45 min in a CEM Discover microwave reactor. The reaction mixture was filtered, and the precipitate was washed with water.

### 3.3. Sample Preparation

All compounds were dissolved with DMSO 100% to obtain a stock solution of 10 mM. In all experiments, the stock solution was employed to obtain a final concentration diluted in Krebs Henseleit solution (vascular reactivity assay) and DMEM supplemented with 10% FBS (cytotoxicity assay).

### 3.4. Animals

Male Wistar normotensive rats (*Rattus norvegicus*) weighing between 200 and 300 grams were supplied by the Central Animal Facility of Fluminense Federal University (NAL-UFF) (Rio de Janeiro, Brazil). The animals were kept in standard environmental conditions of temperature (22 ± 1 °C) and 12 h light/dark cycle, with free access to food (Purina, Brazil) and water. All experiments were conducted in accordance to the guidelines established by National Council for Animal Experimentation Control (CONCEA, Brazil). This study was approved by the Ethics Committee in the Use of Animals of the Fluminense Federal University (CEUA-UFF), certificate under protocol number: 795/2017.

### 3.5. Compounds and Solution

L-phenylephrine hydrochloride (Phe), acetylcholine chloride (Ach) and dimethyl sulfoxide (DMSO) were purchased from Sigma-Aldrich (São Paulo, Brazil). Reagents employed in the preparation of Krebs Henseleit solution were all obtained from Merck (Rio de Janeiro, Brazil).

### 3.6. Vascular Reactivity

Animals were anesthetized by isoflurane (1% mg/kg) inhalation and, after the loss of motor control, rats were euthanized by cervical dislocation. Subsequently, the thoracic aorta was dissected and cleaned of connective tissue to obtain rings 4 mm in length that were suspended in a 15 mL organ bath (Panlab four chamber organ bath, AD-Instruments, Sydney, Australia). Tissues were kept in Krebs Henseleit solution at 37 °C with the following composition (mM): NaCl 120; KCl 5.0; MgCl_2_ 1.1; CaCl_2_ 2.5; NaH_2_PO_4_ 1.2; N-[2-hydroxyethyl] piperazine-N’-[2-ethane-sulfonic acid] (HEPES) 10; NaHCO_3_ 15; and glucose 11, bubbled constantly with a gas mixture of 95% O_2_ and 5% CO_2_ (pH 7.4). Each arterial segment was suspended between two steel hooks connected to an isometric transducer to measure tension through a data acquisition system (PowerLab 8 and LabChart Pro, AD-Instruments, Australia). After a stabilization period of 60 min at a rest tension of 1.0 g with periodic changes of solution (every 15 min), a stable contraction was achieved with 1 µM Phe. Functional endothelial integrity was assessed by the ability of 10 µM ACh to induce ≥70% relaxation in vessels pre-contracted with 1 µM Phe.

### 3.7. Cytotoxicity

A 3-[4,5-dimehyl-2-thiazolyl]-2,5-diphenyl-2H-tetrazoliumbromide (MTT) assay was employed to assess the general cytotoxicity for 4-phenylquinazolin-2(1H)-one derivatives. VERO cells (African Green monkey (*Cercopithecus aethiops*) kidney cell line) were used and obtained from the Rio de Janeiro Cell Bank (PABCAM, Federal University, Rio de Janeiro, Brazil) and grown in Roswell Park Memorial Institute medium (Sigma-Aldrich, St. Louis, MO, USA) supplemented with 10% heat-inactivated fetal bovine serum (FBS; Gibco BRL, Gaithersburg, MD, USA), without antibiotics. Cells were plated in plastic Petri dishes and incubated at 37 °C under a 95% O_2_/5% CO_2_ atmosphere. Cell culture was maintained up to 15 days until cells reached 90% confluence.

The MTT cell viability assay investigates the number of viable cells by measuring the mitochondrial-dependent reduction of water-soluble tetrazolium into an insoluble formazan product. The absorbance obtained is directly proportional to the activity of functional mitochondria and represents the number of viable cells. Briefly, VERO cells were seeded at a density of 1 × 10^4^ cells per well in 96-well plates containing 200 μL of DMEM culture medium supplemented with 10% FBS and incubated at 37 °C and 5% CO_2_ atmosphere. After the first 24 h, either vehicle only (control) or compounds (0.3, 1, 3, 10 and 30 μM) were added to the culture medium and VERO cells were incubated for an additional 48 h. At the end of the treatment period, the medium was replaced with complete fresh medium containing 5 mg/mL tetrazolium dye (MTT) and incubated for 3 h; then, the medium was removed and 100 μL of DMSO was added. Absorbance was measured with a plate reader (SpectraMax 190, CA, USA) at a wavelength of 550 nm and expressed as a percentage of the control (vehicle).

### 3.8. Expression of Data and Statistical Analysis

Values are expressed as mean ± standard error of mean (SEM). A *P*-value less than 0.05 was termed statistically significant. The IC_50_ values (defined as the concentration of the test compound that reduced 50% of the maximal contraction) were obtained by actual concentration-response curve fitting using GraphPad Prism 6.0 software (GraphPad Software, San Diego, CA). Statistical significance was determined using Bonferroni´s multiple comparison for vascular reactivity experiments and using Turkey´s multiple comparison for cytotoxicity evaluation following two-way analysis of variance (ANOVA).

## 4. Conclusions

All tested compounds were obtained through simple synthetic reactions using simple methodologies. The reaction methodology using microwave irradiation provided better yields and lower reactional times when compared with the traditional methodology. In vitro screening for a relaxant effect on aorta rings isolated from rats showed that compounds **2a**–**f** exhibited significant concentration-dependent effects that were dependent on the presence of an intact endothelium, suggesting a mechanism of action mediated by endothelium-derived factors (EDFs). Additionally, only one of the active compounds exhibited significant cytotoxicity at the highest concentration employed, suggesting low toxicity. Nevertheless, further toxicity investigations are required to ensure these findings. In conclusion, this series of molecules displayed the potential to serve as templates for the development of new drugs for diseases involving vascular smooth muscle, especially in disorders in which endothelial dysfunction is a relevant pathophysiological feature.

## Figures and Tables

**Table 1:** Optimization of reaction conditions for the synthesis of compounds **2a**–**f**.

Compound	Method	Yield (%)	Time
**2a**	Conventional	78	18 h
**2a**	MW	92	30 min
**2b**	Conventional	16	30 h
**2b**	MW	31	45 min
**2c**	Conventional	67	24 h
**2c**	MW	89	30 min
**2d**	Conventional	78	22 h
**2d**	MW	80	30 min
**2e**	Conventional	68	24 h
**2e**	MW	63	30 min
**2f**	Conventional	85	28 h
**2f**	MW	88	30 min

**Table 2:** Compounds identification (ID), molecular weight and IC_50_ vasorelaxation response in endothelium-intact (E+) aorta arterial vessels.

Compound ID	R_1_	R_2_	R_3_	MW (g/mol)	IC_50_ (µM)
**2a**	H	H	H	222.24	39.72 ± 6.77
**2b**	NO_2_	H	H	267.24	16.39 ± 3.50
**2c**	Cl	Me	H	270.71	4.31 ± 0.90
**2d**	Cl	F	H	274.68	26.53 ± 8.98
**2e**	Br	H	H	301.13	4.94 ± 1.21
**2f**	Cl	H	Me	270.71	3.41 ± 0.65
